# Correction

**Published:** 1850-10

**Authors:** 


*Correction.*—At the meeting of the American Society of Dental Surgeons, held at Saratoga, August, 1848, Dr .C. C. Allen sent in his resignation of membership, and, as is usual in cases where the dues have not all been paid, and in accordance with a requirement of the constitution, a resolution was offered and passed to the effect that the resignation be accepted, when his dues were paid. Now, in the case of Dr. A., there were no dues owing to the society. They had all been paid up to that time, and the resolution must have been offered and passed without any reference having been made to the book of the treasurer. This statement is due to Dr. Allen, and we trust it will correct any false impression that may have been made by the published proceedings of the meeting above referred to.

